# Supplementary figures and images for: Comprehensive Genome-Wide Analyses of Poplar R2R3-MYB Transcription Factors and Tissue-Specific Expression Patterns under Drought Stress
Source: Int J Mol Sci. 2023 Mar 11;24(6):5389. doi: 10.3390/ijms24065389 (PMC10049292; doi:10.3390/ijms24065389)

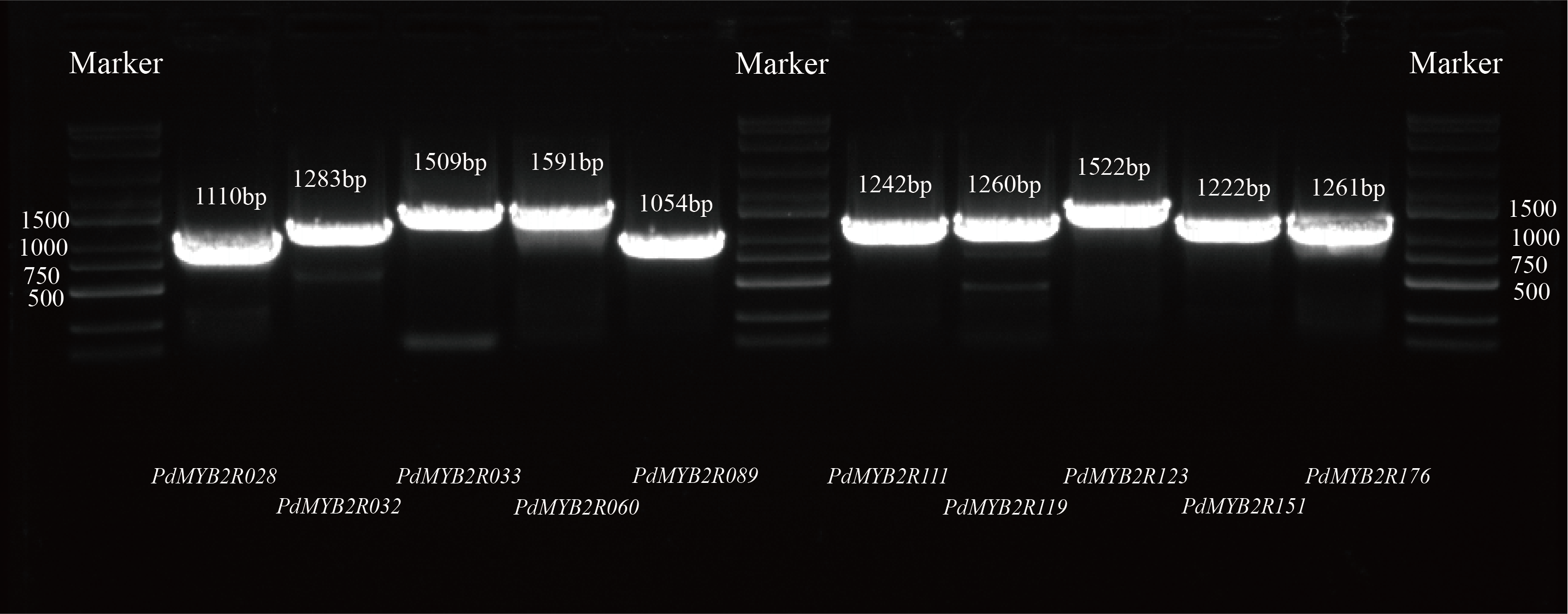

Supplement: Supplementary file 1 [file ijms-24-05389-s001.zip › Figure S1.png]

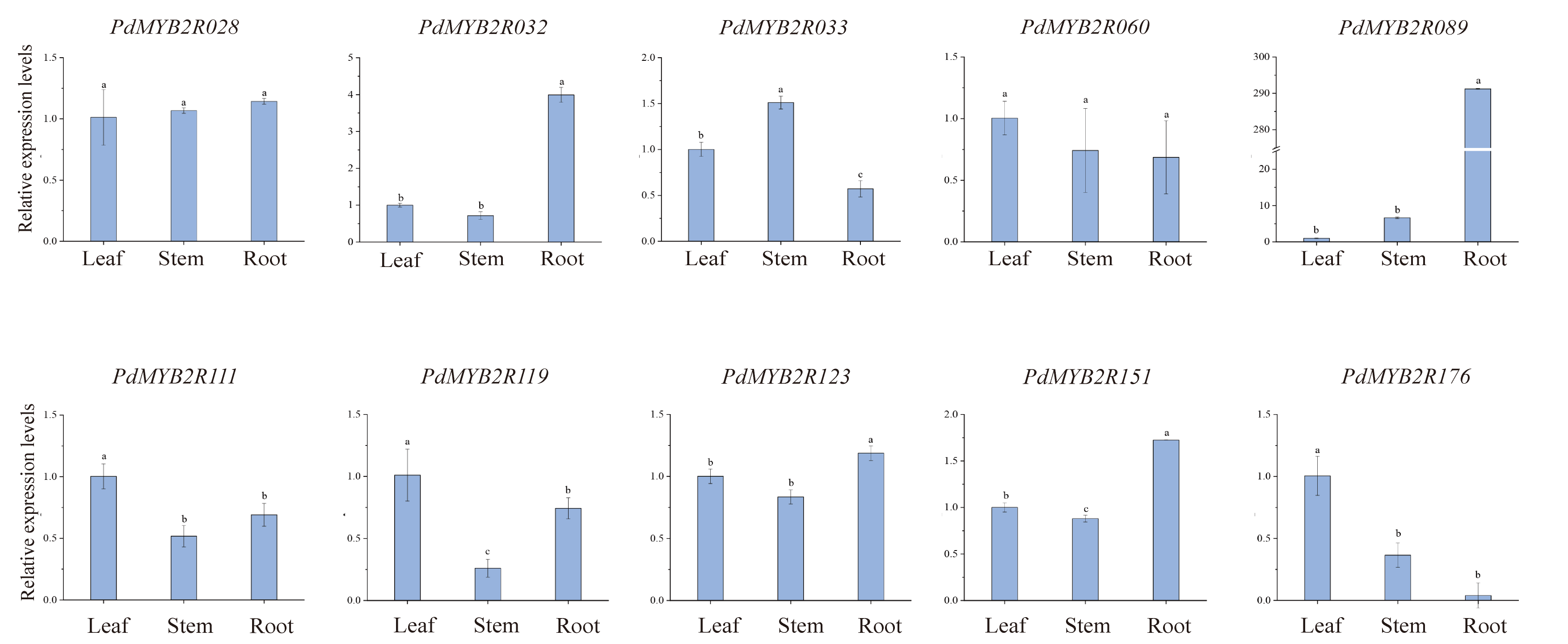

Supplement: Supplementary file 1 [file ijms-24-05389-s001.zip › Figure S2.png]
